# White Meat Consumption and Cardiometabolic Risk Factors: A Review of Recent Prospective Cohort Studies

**DOI:** 10.3390/nu14245213

**Published:** 2022-12-07

**Authors:** Evangelia Damigou, Rena I. Kosti, Demosthenes B. Panagiotakos

**Affiliations:** 1 Department of Nutrition and Dietetics, School of Health Science and Education, Harokopio University, 176 76 Athens, Greece; 2 Department of Nutrition and Dietetics, School of Physical Education, Sports and Dietetics, University of Thessaly, 382 21 Trikala, Greece

**Keywords:** white meat consumption, cardiometabolic risk factors, CVD risk factors

## Abstract

Although the association between meat consumption and cardiovascular diseases (CVDs) has been extensively investigated, studies focusing specifically on the relationship between white meat consumption and CVD risk factors are fewer with controversial findings. The aim was to evaluate the relationship between white meat consumption and the incidence of cardiometabolic risk factors. A comprehensive literature search of PubMed articles was conducted from 2010 to 2022 (1 November), according to PRISMA (Preferred Reporting Items for Systematic reviews and Meta-Analyses) guidelines. Thirteen prospective cohort studies were selected studying mainly poultry, with the exception of one study that also analyzed rabbit meat. From the seven studies on the risk of type 2 diabetes mellitus, four studies found no association, two studies found positive associations, and two studies found inverse associations when comparing poultry to other meats. Of the two studies on the risk of hypertension, one observed no association and one a positive association. Of the two studies on weight management, one observed a positive association with weight gain, the other study observed the same relationship only for chicken with skin, while for chicken without skin a positive relationship with relative weight loss was found. As for metabolic syndrome and its components, two studies revealed inverse associations with white meat intake. Only fresh lean white meat consumption seems to have potential beneficial effects on cardiometabolic risk factors. Future research should scrutinize consumption habits related to white meat intake when investigating its association with cardiometabolic risk factors.

## 1. Introduction

A rise in the previously declining age-standardized rate of cardiovascular disease (CVD) that has been observed might be attributed to the increasing prevalence of CVD risk factors such as type 2 diabetes mellitus, hypertension, hypercholesterolemia, and obesity [1]. International dietary guidelines [2] as well as the Sustainable Development Goals [3] recommend a reduction of meat consumption and a better adherence to a more plant-based diet; however, meat consumption is still high, especially in the Western world or for people adopting a westernized diet. With the exception of some African and Asian territories, in the majority of the world, daily meat consumption is expected to be over 165 g per day per person in 2030 [4,5], i.e., numbers which far surpass the recommended cooked meat consumption of 350–500 g per week [6]. 

Beyond the serious environmental concerns of the increased meat consumption [7], a lot of studies have been conducted relevant to the association between meat consumption and health outcomes. Specifically, meta-analyses have shown controversial results highlighting the need for differentiation between the types of meat consumed and their association with CVD risk. For instance, in a recently conducted meta-analysis, a reduction of processed as well as unprocessed red meat consumption was associated with small reductions in risk for cardiovascular mortality, stroke, myocardial infarction (MI), and type 2 diabetes mellitus (T2DM) albeit the evidence was of low certainty [8]. 

Other meta-analyses have shown a robust positive association between red and processed meat consumption and incidence of CVD and diabetes [9,10]. On the contrary, white meat has recently been proposed as a potential healthier alternative to red meat showing a neutral association with CVD mortality and morbidity [11]. To the best of our knowledge, studies focusing specifically on the relationship between white and in particular lean meat consumption and CVD risk factors are fewer with unclear findings.

Thus, the aim of this study was to review the relationship between white meat consumption and cardiometabolic risk factors based on recent prospective cohort studies; we hypothesized that lean white meat is beneficial for reducing the incidence of cardiometabolic risk factors. 

## 2. Methods

### 2.1. Search Strategy

A comprehensive literature search of PubMed articles was carried out from 1 January 2010 to 1 November 2022, according to PRISMA (Preferred Reporting Items for Systematic reviews and Meta-Analyses) guidelines [12]. Using appropriate Boolean operators (AND, OR, and NOT), the key words used in the search string were (“poultry” or “white meat” or “chicken” or “turkey” or “rabbit”) and (“hypertension” OR “diabetes mellitus” OR “obesity” OR “hypercholesterolemia” OR “triglycerides” OR “non-alcoholic fatty liver disease” OR “metabolic syndrome”) and (“meat” or “consumption” or “intake” or “serving”). Two review authors independently extracted all data and a third author resolved disagreements.

**Figure 1:**
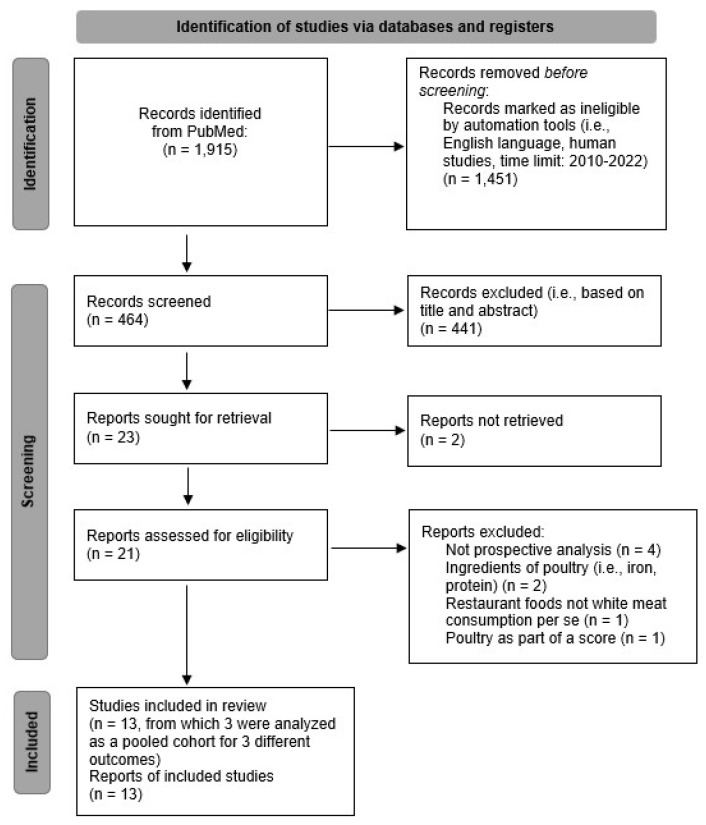
The flow diagram of study identification, screening, and inclusion process based on PRISMA guidelines [13].

The flow-chart regarding the details of the literature search is reported in Figure 1; from a total of 1915 reports found through the search string, 464 remained after the use of filters according to inclusion and exclusion criteria (i.e., humans, adult: 19+ years, language: English, publication date: 2010–2022). To search for relevant studies, a hierarchical approach was used (i.e., screening the title or abstract for the 464 records, choosing 23 records for retrieval and reading the full-text manuscript of 21 articles that were successfully retrieved). References of retrieved articles were also manually searched, but no additional studies that matched the criteria were found.

### 2.2. Inclusion and Exclusion Criteria

The CoCoPop (condition, context, and population) [14] method was used to formulate and narrow the focus of the research question (Is white meat consumption of healthy adults associated with the occurrence of cardiometabolic risk factors?); thus, inclusion and exclusion criteria were formulated, as shown in Table 1. 

### 2.3. Data Collection

Key information manually extracted from the 13 selected studies and then displayed on an Excel spreadsheet included: “Author and Publication Year”, “Study name”, “Country”, “Length of follow-up”, “Number of participants”, “Sex”, “Age”, “Dietary assessment method”, “White meat type”, “Comparison group”, “CVD risk factor”, “Findings”, and “Adjustments” as shown in Table 2.

## 3. Results

### 3.1. Study Characteristics

A total of 13 prospective cohort studies were selected to be included in the present review. Details concerning all of the above studies are shown in Table 2. Included studies focused on white meat consumption usually referred to as “poultry intake” (i.e., chicken, turkey). No studies relevant to the risk of non-alcoholic fatty liver disease, matching the inclusion criteria, were found.

From the 13 studies found, 7 addressed the risk of type 2 diabetes mellitus [15,16,17,18,19,20,21], 2 investigated the risk of hypertension [22,23], 2 studied the risk of weight gain or change [24,25], and 2 evaluated metabolic syndrome and its six components (i.e., systolic blood pressure (SBP), diastolic blood pressure (DBP), high density lipoprotein (HDL), triacyclglycerols (TAG), fasting glucose, waist circumference) [26,27]. Of note, 3 of the 13 studies were a pooled analysis of the Nurses’ Health Study (NHS), Nurses’ Health Study II (NHS II), and Health Professionals Follow-up Study (HPFS) cohorts, but each study investigated a different CVD risk factor outcome; therefore, all 3 were included [21,22,25]. Across the studies, time to follow-up ranged from 2 [27] to 24 years [25]. The minimum number of participants was 1126 [27] and the maximum number was 461,036 [20]. Finally, seven of the included studies evaluated white meat intake more than one time, via FFQs, making the evaluation of white meat consumption in these studies more representative of the populations’ actual intake [17,20,21,22,23,25,26].

### 3.2. Participants’ Characteristics

The participants of the included studies were healthy adults of the general population without CVD or CVD risk factors (inclusion criterion), of all ages (minimum age: 18 years), and with the exception of one study (i.e., men: 73%) [27], in all other included studies, the majority of the participants was women. Finally, the included populations were mostly from the US [15,21,22,25,27] and Asia [17,18,20,23,26], followed by Europe [16,19,24]. 

### 3.3. White Meat and CVD Risk Factors

#### 3.3.1. White Meat and Diabetes Mellitus

From the seven studies addressing the risk of type 2 diabetes mellitus that were found [15,16,17,18,19,20,21], four studies found no association of poultry intake and the risk of T2DM [15,16,17,20], while two studies reported positive associations; the study conducted by Steinbrecher et al. [15] observed that processed poultry intake (5th vs. 1st quintile) was associated with an increased risk of T2DM by 23–30% (HRmen: 1.30, 95%CI: 1.17, 1.44; HRwomen: 1.23, 95%CI: 1.10, 1.38). The other study showed that poultry intake (4th vs. 1st quartile) was associated with 15% higher risk of T2DM (HR: 1.15, 95% CI: 1.06, 1.24, P for trend = 0.004); moreover, this association was mediated by heme iron intake [18]. On the contrary, when comparing white meat, namely poultry, to other meats, inverse associations were found in two studies [19,21]. One study concluded that when replacing 150 g/week of processed red meat with poultry, the risk of T2DM was reduced by 4% (HR: 0.96, 95% CI 0.93, 0.99), but no association was observed when poultry intake replaced low fat or high fat red meat [19]. In another study, which included 46,023 women from the Nurses’ Health Study (NHS), 75,196 women from the Nurses’ Health Study II (NHS II), and 27,634 men from the Health Professionals Follow-Up Study (HPFS), during a 4-year period, findings showed that when increasing poultry consumption (per 1 serving/day, i.e., serving size range: 112–168 g) while concomitantly decreasing the intake of red meat, 18% lower risk of T2DM in the subsequent 4-year period was observed (pooled HR: 0.82, 95% CI: 0.75, 0.90) [21]. 

#### 3.3.2. White Meat and Hypertension

Regarding the association between white meat consumption and the risk of hypertension, a prospective study from Tehran Lipid and Glucose Study found no association of poultry intake and the 3-year incidence of hypertension when poultry was studied either as a continuous variable (i.e., per 1 g/d) or as a categorical one (3rd vs. 1st tertile) [23]. However, another study with 188,518 non-hypertensive individuals from the NHS, NHS II, and HPFS reported that the highest (≥1 servings/day, serving size range: 112–168 g) compared to the lowest (<1 serving/month) category of poultry intake was positively associated with risk of hypertension (pooled HR: 1.22, 95%CI: 1.12, 1.34; *p*-trend < 0.001), although this association was null in the subgroup of women from the NHS [22].

#### 3.3.3. White Meat and Weight Management

Regarding the association of white meat with weight management, one study found a positive association between poultry intake and weight gain [24]. Another study with 120,784 participants free of chronic disease or obesity at baseline from the NHS, NHS II, and HPFS, studying chicken with and without skin (per increase of 1 serving/day, serving size range: 112–168 g), concluded that consumption of chicken with skin was positively associated with long-term weight gain of approximately +0.5 kg every 4 years, while consumption of chicken without skin was positively associated with a relative weight loss of approximately −0.5 kg every 4 years [25].

#### 3.3.4. White Meat and Metabolic Syndrome

Finally, two studies investigating the association between metabolic syndrome (MetS) and its six components (i.e., fasting glucose, SBP, DBP, HDL, TAG, waist circumference) and white meat consumption [26,27] found inverse associations. Specifically, Hajihashemi et al. [26] found that an increase in the frequency of poultry intake was inversely associated with the risk of metabolic syndrome in multi-adjusted models (OR 0.78; 95% CI: 0.72–0.85) in 6504 adults free of MetS at baseline prospectively studied for a median of approximately 11 years. Moreover, Riseberg et al. [27] concluded that unprocessed poultry intake was inversely associated with TAG (b: 24.5, SE: 9.13) but not with other CVD risk factors (i.e., fasting glucose, SBP, DBP, HDL, waist circumference) in 1126 participants followed for 2 years.

## 4. Discussion

Meat consumption is one of the major modifiable food groups that affect disease risk, thus it is of the greatest importance to identify the type of meat that could decrease the risk of morbidity and mortality of CVD. From the present review, it seems that the adverse health effects associated with red and processed meat consumption are not shared by white meat and in particular from lean unprocessed white meat. Poultry consumption does not seem to affect the incidence of type 2 diabetes, and thus it is prudent for white meat to be consumed as a replacement of red or processed meat. Although poultry consumption was associated with a negative effect on weight loss, lean cuts were proven to be beneficial for weight management. Poultry consumption is beneficial for preventing metabolic syndrome or reducing TAG. Lastly, the association between white meat consumption and hypertension ranges from neutral to aggravating, which could be attributed to potential confounding factors [28]. 

Indeed, multiple confounding factors may have affected the aforementioned relationships. The most important factors that were pinpointed in the reviewed studies are the type of white meat consumed (processed or with the skin vs. unprocessed or without the skin) as well as the type of food replaced by white meat. For instance, as shown in a study [25], eating chicken with or without skin might “transform” chicken consumption from harmful (with skin) to beneficial (without skin) for long-term weight management. Of note, chicken skin is high in saturated fat, possibly explaining the observed difference [29]. Additionally, in the study conducted by Steinbrecher et al. [15] on 75,512 participants of the multiethnic cohort (i.e., Caucasians, Japanese Americans, and Native Hawaiians), with a median follow-up of 13.5 years, only the consumption of processed poultry was positively associated with type 2 diabetes incidence and not that of fresh poultry. Population characteristics such as age, sex, ethnicity/nationality, as well as the baseline diet or the quantity of the white meat consumed might also play a role. For instance, there was no uniformity of serving size, with serving size ranging from 98 g to 168 g between the included studies [21,22,25,27,30,31]. Furthermore, study characteristics such as the diagnostic criteria of each cardiometabolic outcome or the time to follow-up might have influenced the results as well.

Certain meat ingredients (i.e., heme iron, saturated fat) might explain the association between white meat and cardiometabolic risk factors. For instance, heme iron has been shown to be detrimental for CVD risk and mortality, but compared to red meat, white meat has lower concentrations of heme iron [32,33]. Interestingly, in one of the included studies, the harmful relation between poultry intake and the risk of type 2 diabetes was completely mediated by heme iron intake [18]. Furthermore, white meat has lower concentrations of saturated fat compared to other meats, especially the lean cuts, and a better lipid profile consisting mainly of unsaturated fatty acids, with the exception of the skin which is full of saturated fat [10,12,34].

In addition, total white meat consumption is usually computed via FFQs and may include different cuts/parts (e.g., chicken breast vs. drumsticks/legs), processed white meat (e.g., turkey sausage), as well as mixed dishes (e.g., chicken/turkey/duck with rice). Therefore, the observed harmful association between white meat consumption and certain cardiovascular risk factors [18,22,24] could be attributed to the lack of differentiation regarding parts/cuts consumed (skin or without skin), kind of white meat consumed (processed vs. unprocessed), or cooking method employed (fried or grilled). Indeed, foods are rarely eaten alone; therefore, food ingredients such as refined grains or sources of saturated fat (e.g., butter, cheese, mayonnaise) can be eaten concomitantly with white meat. Thus, one could presume that food combinations with white meat may mask the potential beneficial effects of lean white meat. For instance, common ingredients accompanying white meat, such as refined grain intake (e.g., white rice, white bread), are associated with increased risk of cardiometabolic risk factors [35,36], while vegetable consumption is associated with decreased cardiometabolic risk [37]. 

Concerning cooking methods (i.e., processing, addition of sodium/preservatives or fat sources, cooking temperature) [34], the literature showed that processed meat having a high content of sodium, preservatives, and dietary advanced glycation and lipoxidation end-products [38] affects adversely cardiometabolic risk beyond blood pressure [39,40]. Based on another analysis of a pooled cohort from the NHS, NHS II, and HPFS with 12–16 years of follow-up, a higher frequency of open-flame and/or high-temperature cooking for chicken (as well as red meat) was independently associated with an increased risk of T2DM, weight gain, and obesity [41].

For people consuming high amounts of animal protein, turning to plant-based diets—even though protective for their health—might be humanly impossible. Therefore, white meat such as chicken, turkey, rabbit, duck, and goose might be a good alternative for reducing the elevated cardiometabolic risk that is associated with red and processed meat [8]. This substitution might also be beneficial for planet sustainability. Although sustainable diets are plant-based, animal protein is a part of them. Given that beef, a main animal protein, is the most energy-consuming food to be produced [42], lean white meat could be considered as a prime candidate for the space of animal protein in the planetary health diet [43]. 

### Strengths and Limitations

One of the limitations of this review is that only one database was searched (i.e., PubMed) and the lack of the assessment of study quality (risk of bias). However, a systematic process was used for retrieving studies. To the best of our knowledge, this is the first review analyzing the recent evidence regarding white meat consumption and the occurrence of cardiometabolic risk factors based on prospective cohort studies. 

In addition, this review contains different populations and base diets, and that fact might have influenced the results, which makes these findings more generalizable. Nevertheless, publication bias might have existed [44], and that might point to an over-estimation of the relationship between white meat and CVD risk factors. Finally, an inherent limitation of most prospective cohort studies is the use of FFQs, which entail recall bias. 

## 5. Conclusions

In conclusion, it was observed that only fresh/unprocessed and lean white meat consumption seems to have potential beneficial effects on cardiometabolic risk factors. Future research should scrutinize consumption habits related to white meat intake when investigating its relationship with cardiometabolic risk factors. Therefore, unprocessed lean white meat is a good alternative to red meat and a sustainable prime source of high-quality protein and vitamins.

## Figures and Tables

**Table 1:** Inclusion and exclusion criteria of this review according to the CoCoPop method.

	Inclusion Criteria	Exclusion Criteria
Condition	CVD risk factors (i.e., diabetes mellitus, hypercholesterolemia, hypertension, obesity, metabolic syndrome, non-alcoholic fatty liver disease)	CVD risk
Context	Studies researching only white meat consumption alone or comparing to other foods	Studies assessing white meat consumption as a part of different diets (i.e., Mediterranean, DASH, vegetarian diet). Studies evaluating different ingredients of white meat (i.e., heme iron, animal protein).
Population	General healthy adult population (>18 years old) free of CVD or CVD risk factors	Age: <18 years old Patients with CVD or CVD risk factors
Study type	Only prospective cohort studies (or studies analyzed prospectively)	Other types of epidemiological studies (i.e., case-control, cross-sectional, or experimental) or prospective cohorts studied with such analyses (i.e., case-cohort, retrospective analysis) Reviews Meta-analyses Position papers, editorials
Time-limit	From 2010 up to 2022 (recent studies)	Articles published before 2010
Language restriction	Only articles published in English	Articles published in other languages

Abbreviations: CVD: cardiovascular disease.

**Table 2:** Study characteristics of recent prospective cohort studies included in this review.

Author, Publication Year	Study Name	Country	Length of Follow-Up (Years)	N (Total)	Men, %	Age (Years Old)	Dietary Assessment Method	White Meat Type	Comparison Groups	CVD Risk Factor (n Cases)	Findings	Adjustments
Type 2 diabetes mellitus												
Steinbrecher et al., 2011 [15]	The Multiethnic Cohort	USA	median: 13.5	75,512 participants (29,759 Caucasian, 35,244 Japanese American, 10,509 Native Hawaiian participants)	48	Range: 45–75	FFQ (designed for this population) (at baseline)	Fresh poultry and processed poultry	Quintiles: 5th vs. 1st quintile (g/d)	T2DM (n = 8587 cases)	(a) Fresh poultry intake: no association with risk of T2DM (HR men: 1.06, 95%CI: 0.96, 1.18; HR women: 1.01, 95%CI: 0.90, 1.14). (b) Processed poultry intake: increased risk of T2DM by 30% for men and 23% for women (HRmen: 1.30, 95%CI: 1.17, 1.44; HR women: 1.23, 95%CI: 1.10, 1.38).	Ethnicity, education, BMI, physical activity, total energy intake (log-transformed) (stratified by age at cohort entry).
van Woudenbergh et al., 2012 [16]	The Rotterdam Study	Netherlands	Median: 12.4	4366 participants	40	Mean (SD): 67.3	(a) Self-administered questionnaire (2 times/month), (b) Semiquantitative FFQ (at baseline)	Poultry	Highest vs. lowest intake (>18 vs. 0 g/d).	T2DM (n = 456 cases)	Poultry intake: no association with the incidence of T2DM (RR: 0.95, 95%CI: 0.74, 1.22).	Age, sex, smoking, diet prescription, family history of diabetes intake of energy, energy-adjusted carbohydrates, energy-adjusted polyunsaturated fatty acids, energy-adjusted fiber, energy-adjusted milk, energy adjusted cheese, soya, fish, alcohol, tea, intake of red meat and processed meat.
Kurotani et al., 2013 [17]	Japan Public Health Center-based Prospective Study	Japan	5	63,849 participants	43	Range: 45–76	FFQs (at baseline, second and third surveys)	Poultry (i.e., grilled chicken, deep-fried chicken)	Quartiles: 4th vs. 1st quartile (g/d)	T2DM (n = 1178 cases)	Poultry intake: no association with T2DM (HR men: 1.03, 95%CI: 0.81, 1.30; HR women: 0.97, 95%CI: 0.74, 1.27).	Age, public health center area, BMI, smoking status, alcohol consumption, total physical activity, the history of hypertension, coffee consumption, the family history of diabetes, Mg intake, Ca intake, rice intake, fish intake, vegetable intake, soft drink consumption, energy intake.
Talaei et al., 2017 [18]	The Singapore Chinese Health Study	China	Mean: 10.9	63,257 Chinese adults	42.7	Range: 45–74	Semiquantitative FFQ (at baseline)	Poultry	Quartiles: 4th vs. 1st quartile (g/d)	T2DM (n = 5207 cases)	Poultry intake: 15% higher risk T2DM (HR: 1.15, 95% CI: 1.06, 1.24, P for trend = 0.004). This association was mediated completely by heme iron intake (HR: 1.01, 95%CI: 0.91, 1.12, P for trend: 0.973).	Age, sex, dialect, year of interview, educational level, body mass index, physical activity level, smoking status, alcohol use, baseline history of self-reported hypertension, adherence to the vegetable-, fruit-, and soy-rich dietary pattern, total energy intake (+heme iron intake for mediation analysis).
Ibsen et al., 2019 [19]	Danish Diet, Cancer and Health study	Denmark	Median: 15.4	53,163	46.8	Range: 50–64	FFQ (at baseline)	Poultry (i.e., turkey and chicken: meat and skin)	g/week	T2DM (n = 6879 cases)	Poultry intake: (a) When replacing 150 g/week of processed red meat: inverse association with T2DM (HR: 0.96, 95% CI 0.93, 0.99). (b) When replacing 150 g/week of low fat or high red fat meat: no association.	Sex, date of enrolment, baseline age, total energy intake, smoking status, alcohol intake, physical activity and level of education, whole grains, fruits, vegetables, dairy products, potatoes, fatty potatoes and soft drinks, body mass index, waist circumference, BMI.
Du et al., 2020 [20]	A prospective cohort study of the China Kadoorie Biobank	China	9	461,036 adults free of CVD, diabetes or cancer	41	Mean: 51	A validated interviewer-administered laptop-based questionnaire (at baseline and re-surveys)	Poultry (i.e., chicken, duck, goose) (baseline and usual consumption)	(days/week, g/d)	T2DM (n = 14,931 cases)	Per 50 g/day of poultry intake (usual consumption): no association with T2DM (HR: 0.96, 95% CI: 0.83, 1.12).	Age at risk, sex and region, education, income, smoking, alcohol consumption, physical activity, family history of diabetes, fresh fruit consumption, red meat, fish, BMI.
Würtz et al., 2021 [21]	Nurses’ Health Study, Nurses’ Health Study II, and Health Professionals Follow-Up Study	USA	2,113,245 person-years of follow-up	148,853 participants (27,634 males in the HPFS, 46,023 females in the NHS, and 75,196 females in the NHS II).	19	Ranges at baseline: NHS I (women): 30–55, NHS II (women): 25–42, HPFS (men): 40–75.	FFQs (every 4 years)	Poultry (chicken and turkey, with or without skin)	servings/d (serving size range: 112–168 g)	T2DM (n = 8763 cases)	Per 1 serving/d increase in poultry, concomitantly with 1 serving/d decrease in intake of red meat, during a 4 year period: lower risk of T2DM in the subsequent 4-year period (pooled HR: 0.82, 95% CI: 0.75, 0.90)	Protein foods, age, calendar time, calories (initial and change), marital status, race, family history of diabetes, history of hypertension, history of hypercholesterolemia, BMI, alcohol intake (initial and change), modified AHEI (initial and change), smoking status change, physical activity (initially and change) and for women initial menopausal status and use of postmenopausal hormones, initial intake of red meat, poultry, seafood, low-fat dairy, high-fat dairy, eggs, legumes, nuts, simultaneous weight change.
Hypertension												
Borgi et al., 2015 [22]	Nurses’ Health Study, Nurses’ Health Study II, and Health Professionals Follow-Up Study	USA	2,936,359 person-years of follow-up	188,518 non-hypertensive individuals (NHS I, n = 62,273 women, NHS II, n = 88,831 women, HPFS, n = 37,414 men).	20	ranges at baseline: NHS I (women): 30–55, NHS II (women): 25–42, HPFS (men): 40–75.	FFQs (every 4 years)	Poultry (chicken and turkey, with or without skin)	Highest vs. lowest intake (≥1 servings/dvs. <1 serving/month) (serving size range: 112–168 g)	HTN (n = 78,208 cases)	(a) Poultry intake (Highest vs. lower category): positive association with risk of HTN (pooled HR: 1.22, 95%CI: 1.12, 1.34; p-trend < 0.001). (b) NHS I (women), higher poultry intake: no association. NHS II (young women), higher intake of poultry: positive association. HPFS (men), greater poultry consumption: positive association. (c) Similar results when all types of animal flesh were used in the multi-adjusted analyses (Highest vs. lowest: HR: 1.12, 95%CI: 1.02, 1.23; per serving/day: HR: 1.07, 95%CI: 1.03, 1.10)	Age, race/ethnicity, BMI, current smoking status, physical activity, weight change per FFQ cycle, post-menopausal status, alcohol intake, current oral contraceptive use, family history of hypertension, total energy intake, total fruits, vegetables, and whole grains, sugar-sweetened beverage intake, artificially-sweetened diet beverage intake, analgesic use.
Golzarand et al., 2016 [23]	A Prospective Study From Tehran Lipid and Glucose Study	Iran	3	1152 healthy adults	42.3	Mean (SD): 36.0 (11.2)	Semiquantitative FFQ (at baseline and after 3 years)	Poultry (i.e., chicken, turkey)	Tertiles: 3rd vs. 1st tertile (g/d)	HTN (n = 114 cases)	(a) Poultry intake: (3rd vs. 1st): no association with the risk of 3-year incidence of HTN (OR: 1.27, 95%CI: 0.74,2.17). (b) Per 1 g/d: no association with the 3-year incidence of HTN (OR: 1.00, 95%CI: 0.99, 1.001)	Age, sex, BMI, 3-year weight changes, smoking, baseline SBP and DBP, physical activity, dietary intake of energy, sodium, potassium, and fiber.
Obesity and weight management												
Vergnaud et al., 2010 [24]	European Prospective Investigation into Cancer and Nutrition-Physical Activity, Nutrition, Alcohol, Cessation of Smoking, Eating Out of Home and Obesity (EPIC-PANACEA) project.	Europe (10 European countries)	on average: 5	373,803 subjects	28	range: 25–70	Country-specific validated questionnaires (i.e., self-administered quantitative dietary questionnaire, FFQs, combined dietary methods) (at baseline)	Poultry (i.e., chicken, turkey, rabbit)	kcal/d	Weight gain	Poultry intake (per 100 kcal increase): positive association with weight gain (b: 45, 95%CI: 29, 62, *p* < 0.0001).	Sex, age, an indicator of meat consumption, educational level, physical activity level, smoking status, initial BMI, follow-up time, total energy intake, energy from alcohol, plausible total energy intake reporting, dietary factors 1 and 2 derived from maximum likelihood factor analysis (labeled “prudent pattern” and “fresh meat”).
Smith et al., 2015 [25]	Nurses’ Health Study, Nurses’ Health Study II, and Health Professionals Follow-Up Study	USA	range: 16–24	120,784 participants free of chronic disease or obesity at baseline. (46,994 in the NHS, 47,928 in the NHS II, and 25,862 in the HPFS)	21	mean (SD): NHS (women): 48.9 (2.7); NHS II (women): 37.7 (3.2); HPFS (men): 47.3 (2.7).	FFQs (every 4 years)	Poultry (chicken and turkey, with or without skin)	servings/d (serving size range: 112–168 g)	Long-term weight change	(a) Chicken with skin (per increase of 1 serving/d): positive association with long-term weight gain of +0.48 kg (95%CI: +0.06 kg, +0.90 kg) every 4 years. (b) Chicken without skin (per increase of 1 serving/d): positive association with relative weight loss of −0.48 kg (95% CI: −0.70 kg, −0.27 kg) every 4 years.	Age, baseline (of each 4-year period) BMI, sleep duration, change in smoking status, physical activity, television watching, alcohol consumption, fruit intake, vegetable intake, glycemic load, diatary factos (i.e., regular hamurger, lean hambutger, hot dogs, pork as a main dish, bacon, beef/lamb/pork as a mixed dish, deli/sandwich meat, beer/lamb as a main dish, chicken with or without skin, seafood, butter, regular cheese, low-fat milk, whole milk, eggs, low-fat cheese, flavored sweetened yoghurt, plain or artificially sweetened yoghurt, legumes, peanuts, peanut butter, walnuts, other nuts.
Metabolic syndrome and its components												
Hajihashemi et al., 2021 [26]	Isfahan Cohort Study (ICS)	Iran	median: 11.25	6504 adults free of MetS at baseline	49	>35 years	FFQs (at 3 phases)	Poultry	Frequency of consumption (daily, weekly, monthly)	MetS (n = 1869)	Frequency of consumption of poultry: inverse association with risk of MetS (crude: OR 0.73; 95%:CI: 0.68, 0.78) (multiadjusted: OR 0.78; 95% CI: 0.72–0.85).	Age, sex, physical activity, current smoker, BMI, fruits, vegetables, cereal, protein sources.
Riseberg et al., 2022 [27]	Boston Puerto Rican Health Study	USA	2	1126	73	range: 45–75, median (IQR): 56 (51, 63)	Semiquantitative FFQ (at baseline)	Unprocessed white meat (i.e., chicken, turkey).	servings/d (mean ± SD intake of fried chicken: 101 ± 4, other chicken: 98 ± 5)	Cardiometabolic risk factors (i.e., the six components of MetS)	Unprocessed poultry intake: inverse association with TAG (b: 24.5, SE: 9.13). No associations with other factors (i.e., fasting glucose, SBP, DBP, HDL, waist circumference).	Total energy intake, sex, age, education, baseline outcome, smoking, alcohol intake, physical activity, psychological acculturation, fruit and vegetable intake score, omega-3 fatty acid intake, whole grain intake, medication (for blood pressure, triglycerides, and glucose only), and sodium intake (for blood pressure only).

Abbreviations: 95%CI: 95% confidence interval, b: b coefficient, BMI: body mass index, DBP: diastolic blood pressure, FFQs: food frequency Questionnaire, HDL: high-density lipoprotein, HPFS: health professionals follow-up study, HR: hazard ratio, HTN: hypertension, MetS: metabolic syndrome, NHS: Nurses’ Health Study, NHS II: Nurses’ Health Study II, OR: odds ratio, RR: relative risk, SBP: systolic blood pressure, SD: standard deviation, SE: standard error, T2DM: type 2 diabetes mellitus, TAG: triacylglycerols, vs.: versus.

## Data Availability

No applicable.
